# Chronic hyperglycemia before acute ischemic stroke impairs the bilateral cerebrovascular response to exercise during the subacute recovery period

**DOI:** 10.1002/brb3.1990

**Published:** 2020-12-08

**Authors:** Carolyn S. Kaufman, Stephen X. Bai, Sarah M. Eickmeyer, Sandra A. Billinger

**Affiliations:** ^1^ Department of Molecular and Integrative Physiology University of Kansas Medical Center Kansas City KS USA; ^2^ Department of Physical Therapy and Rehabilitation Science University of Kansas Medical Center Kansas City KS USA; ^3^ Department of Physical Medicine and Rehabilitation University of Kansas Medical Center Kansas City KS USA; ^4^ Department of Neurology University of Kansas Medical Center Kansas City KS USA

**Keywords:** cerebrovascular response, Exercise, hemoglobin A1c, middle cerebral artery velocity, stroke

## Abstract

**Background and Purpose:**

Chronic hyperglycemia contributes to cerebrovascular dysfunction by damaging blood vessels. Poor glucose control has been tied to impairments in cerebral blood flow, which may be particularly detrimental for people recovering from major cerebrovascular events such as acute ischemic stroke. In this secondary analysis, we explore for the first time the connection between chronic hyperglycemia before acute stroke and the cerebrovascular response (CVR) to exercise 3 and 6 month into the subacute recovery period.

**Methods:**

We recorded middle cerebral artery velocity (MCAv) using transcranial Doppler ultrasound bilaterally at rest and during moderate‐intensity exercise in stroke patients at 3 (*n* = 19) and 6 (*n* = 12) months post‐stroke. We calculated CVR as the difference between MCAv during steady‐state exercise and resting MCAv. We obtained hemoglobin A1c levels (HbA1c; a measure of blood glucose over the prior 3 months) from the electronic medical record (EMR) and divided participants by HbA1c greater or less than 7%.

**Results:**

Participants with high HbA1c (>7%) at the time of acute stroke had significantly lower CVR to exercise for both the stroke‐affected (*p* = .009) and non‐affected (*p* = .007) hemispheres at 3 months post‐stroke. These differences remained significant at 6 months post‐stroke (stroke‐affected, *p* = .008; non‐affected, *p* = .016).

**Conclusions:**

Patients with chronic hyperglycemia before acute ischemic stroke demonstrated impaired cerebrovascular function during exercise months into the subacute recovery period. These findings highlight the importance of maintaining tight glucose control to reduce morbidity and improve recovery post‐stroke and could have implications for understanding cerebrovascular pathophysiology.

## Graphical abstract

Chronic hyperglycemia in the months before acute ischemic stroke predicts impaired cerebrovascular function during exercise months into the recovery period. This finding highlights the importance of maintaining tight glucose control in patients with diabetes mellitus in order to reduce morbidity and improve recovery after ischemic events.

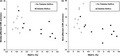

## 1 INTRODUCTION

Chronic hyperglycemia causes systemic vascular damage. (Duckworth et al., 2009; Hemmingsen et al., 2011; Patel et al., 2008) Therefore, the American College of Physicians (ACP) and American Diabetes Association (ADA) recommend long‐term glucose control—specifically, a hemoglobin A1c (HbA1c) level below 7%—in order to minimize severity and frequency of vascular complications in diabetes mellitus (DM) (Qaseem et al., 2018; Targets, 2019). Without adequate control, chronic hyperglycemia increases the risk of stroke and negatively affects cerebrovascular function in other neurological disorders such as Alzheimer's disease and vascular cognitive impairment. (Ergul et al., 2012) Elevated blood glucose causes damage to both large and small vessels with increasing evidence that stroke and vascular cognitive impairment are the result of a combination of pathology in both types of cerebral vessels. (Huber, 2008) Reduced middle cerebral artery velocity (MCAv), a surrogate measure of cerebral blood flow (CBF), has been reported in those with DM at rest and in response to stimuli (e.g., hypercapnia) when compared to control groups. (Cui et al., 2017; Jansen et al., 2016; Kadoi et al.,; Novak et al., 2006) Additionally, we previously reported that cognitively normal older adults with higher cardiovascular risk level, including those with DM, have lower MCAv at rest and in response to moderate‐intensity exercise. (Perdomo et al.,)

In people with stroke, DM is often a comorbid condition along with other traditional cardiovascular risk factors. (Kernan et al., 2014) Two recent reports have shown that people with stroke may have altered cerebrovascular function when compared to their peers. (Kempf & A., Lui, Y., 2019; Robertson et al., 2019) However, specific contributing factors to this cerebrovascular dysfunction, such as chronic hyperglycemia (HbA1c > 7%), have yet to be explored. Thus, it is unknown whether chronic hyperglycemia in the months prior to acute ischemic stroke affects resting and exercising MCAv during the subacute stage of stroke recovery.

To address this gap in the literature, the objective of this secondary analysis was to explore whether elevated HbA1C at the time of acute stroke was associated with impaired MCAv measures during rest and exercise at 3 and 6 months post‐stroke. Specifically, we divided participants into those with chronically uncontrolled blood glucose (defined according to ADA and ACP guidelines as HbA1c > 7%) or controlled blood glucose (HbA1c < 7%) at the time of acute stroke. We hypothesized that the group with elevated HbA1c would have: 1) lower resting MCAv, 2) reduced cerebrovascular response to exercise (CVR, defined as exercising MCAv – resting MCAv), and 3) smaller percent change in MCAv (%ΔMCAv, calculated to control for resting baseline values) from rest to exercise at 3 and 6 months post‐stroke. As an exploratory aim, we evaluated the CVR in participants stratified by Type 2 DM diagnosis with the hypothesis that the CVR would be significantly negatively correlated with HbA1c level for individuals with Type 2 DM.

## 2 METHODS

### 2.1 Participants

This is a secondary analysis from an observational study characterizing MCAv dynamic response to an acute bout of exercise at 3 and 6 months post‐stroke. (Billinger et al., 2005.20100883.) Inclusion and exclusion criteria were covered in detail previously. (Billinger et al., 2005.20100883.) The University of Kansas Medical Center Human Subjects Committee approved all experimental procedures in compliance with the Declaration of Helsinki. Written informed consent was obtained prior to commencement of study procedures. We obtained HbA1c levels from the electronic medical record (EMR) (Epic Systems Corporation, Verona, WI) collected during the acute hospital stay no later than 72 hr after admission to the acute stroke unit at the University of Kansas Health System.

### 2.2 Experimental procedures

The following protocol was performed for Visit 1 (3 months post‐stroke) and Visit 2 (6 months post‐stroke) for each participant and has been described in detail previously. (Billinger et al., 2017; Billinger et al., 2005.20100883.; Kaufman et al., 2019; Kempf & A., Lui, Y., 2019; Ward et al., 2018) The study team obtained the participant's height and weight and calculated body mass index (BMI). (Eknoyan, 2008) A five‐lead electrocardiogram (Cardiocard, Nasiff Associates, Central Square, NY) continuously monitored HR. Beat‐to‐beat blood pressure (BP) was acquired from the left middle finger (Finometer PRO; Finapres Medical Systems, Amsterdam, The Netherlands). (Billinger et al., 2017; Fisher et al., 1985; Kaufman et al., 2019; Kempf & A., Lui, Y., 2019; Ward et al., 2018) A nasal cannula connected to a capnograph (BCI Bapnocheck 9,004) continuously recorded end‐tidal carbon dioxide (P_ET_CO_2_). The sonographer was blinded to side of stroke. Ultrasonic gel was applied to the 2‐MHz transcranial Doppler ultrasound (Multigon Industries, Yonkers, NY) probes and placed over the cranial temporal bone window using established practice standards in positioning and orienting the probe to insonate the MCA. (Alexandrov et al., 2007, 2012) The probes were fixed in place on an adjustable headband.

Each experimental exercise bout consisted of a continuous 7.5‐min recording. We first acquired 90 s of resting data, and then the participant began exercising. Maximum HR (HRmax) was determined by using either: (Brawner et al., 2004).

HRmax = 220 – age, for participants not taking a beta‐blocker, or

HRmax = 164 – (0.72*age), for participants taking a beta‐blocker.

Moderate‐intensity exercise was defined as 45% to 55% of the participant's heart rate reserve. The HR range for moderate‐intensity exercise was determined using the Karvonen formula. (Ferguson,)

HR range = [% exercise intensity*(HRmax – resting HR)] + resting HR.

The participant exercised at moderate‐intensity for 6 min. After the recording ended, the participant sat quietly until HR returned to resting level and then repeated the 7.5‐min experimental bout (90 s of rest and 6 min of exercise), as our previous work showed improved signal‐to‐noise ratio when data acquired from multiple exercise bouts are averaged. (Billinger et al., 2017).

### 2.3 Data acquisition

Raw data acquisition occurred through an analog‐to‐digital unit (NI‐USB‐6212, National Instruments) and custom written software operating in MATLAB (v2014a, The Mathworks Inc. Natick, MA). Sampling of all variables was at 500 Hz and then interpolated to 2.0 Hz. As in our previous work, three‐second averages were calculated and smoothed using a 9‐s sliding window average. (Alwatban et al., 2020; Billinger et al., 2017; Kaufman et al., 2019; Kempf & A., Lui, Y., 2019; Ward et al., 2018) We used R version 3.2.4 (R Core team, Vienna, Austria) with the “nls” function package to model the response. Data with RR intervals > 5 Hz or changes in peak blood flow velocity of > 10 cm/s in a single cardiac cycle were considered artifact and censored. If an acquisition consisted of > 15% censored cardiac cycles, the entire sample was discarded. The cerebrovascular response (CVR) was calculated as the difference between the mean MCAv sampled between minute 3 and 4.5 during steady‐state exercise and the resting MCAv (mean MCAv over the first 90 s of the 8‐min recording). (Kaufman et al., 2019; Kempf & A., Lui, Y., 2019; Sisante et al., 2019) The percent change in MCAv from rest to exercise (%ΔMCAv) was calculated as the CVR divided by resting MCAv multiplied by 100. The CVR and %ΔMCAv were calculated separately for the stroke‐affected and non‐affected sides in each participant.

### 2.4 Statistical analysis

All statistical analyses were performed using SPSS Statistics (IBM). Participants were divided into two groups based on HbA1c value at the time of acute stroke (<7% or >7%). Between‐group differences were assessed using independent *t* tests, Mann–Whitney U tests, or Fisher's exact tests, as appropriate. Data are presented as mean ± standard deviation unless otherwise indicated. We also ran a Pearson's product–moment correlation to characterize the relationship between HbA1c level and CVR for participants diagnosed with Type 2 DM. We set α = 0.05 to protect against type I error.

## 3 RESULTS

Twenty‐six participants with ischemic stroke were enrolled into the parent study. For this secondary analysis, only those data that successfully modeled for bilateral MCAv CVR were used (*n* = 19) at Visit 1 (3‐months post‐stroke). Six participants were lost to follow up, and data for Visit 2 (6‐months post‐stroke) included 12 participants.

### 3.1 Visit 1 (3 months post‐stroke)

Participant demographics and laboratory physiological measurements for Visit 1 are presented in Table 1. Thirteen participants (68%) had HbA1c <7% at the time of acute stroke. There were no between‐group differences for those with low HbA1c (<7%, *n* = 13) and high HbA1c (>7%, *n* = 6) for demographic factors including sex, age, race, BMI, smoking history or hypertension diagnosis. No between‐group differences were found for P_ET_CO_2_ and MAP at rest or during exercise, which is important as both measures can influence MCAv. Contrary to our hypothesis, there were no significant differences in resting MCAv between high and low HbA1c groups on the stroke‐affected (*U* = 53, *z* = 1.228, *p* = .244) or non‐affected (*U* = 56, *z* = 1.491, *p* = .152) side. However, stroke‐affected CVR was lower for the high HbA1c group (mean rank = 5.17) than the low HbA1c group (mean rank = 12.23) (*U* = 10, *z*=−2.543, *p* = .009). Likewise, non‐stroke‐affected CVR demonstrated a lower value for the high HbA1c group (mean rank = 5.00) than the low HbA1c group (mean rank = 12.31) (*U* = 9, *z* = −2.631, *p* = .007). The %ΔMCAv was attenuated for the high HbA1c group for the stroke‐affected (*U* = 10, *z* = −2.543, *p* = .009) and the non‐affected (*U* = 6, *z* = −2.894, *p* = .002) vessels. Table 1 includes data from Visit 1 (3‐months post‐stroke) presented as mean ± standard deviation unless otherwise indicated.

**Table 1:** Demographics and physiological measurements taken at rest and during exercise at 3 months post‐stroke in individuals grouped by Hemoglobin A1c (HbA1c) status at time of acute stroke

	HbA1c < 7% (*n* = 13)	HbA1c > 7% (*n* = 6)	*p*‐value
Sex, *n* male [% male]	8 [62%]	4 [67%]	1.000
Age	59.2 ± 12.3 years	69.8 ± 17.4 years	0.144
Caucasian, *n* [%]	10 [77%]	4 [67%]	1.000
African American, *n* [%]	2 [15%]	3 [50%]	0.262
Native American, *n* [%]	0 [0%]	1 [17%]	0.316
Asian, *n* [%]	1 [8%]	0 [0%]	1.000
Body Mass Index	30.6 ± 6.2 kg/m^2^	27.1 ± 2.0 kg/m^2^	0.085
Current or Past Smoker, *n* [%]	5 [38%]	1 [17%]	0.605
Hypertension	10 [77%]	6 [100%]	0.517
Stroke‐affected resting MCAv	51.1 ± 15.5 cm/sec	59.5 ± 16.1 cm/sec	0.244
Stroke‐affected CVR to exercise	6.8 ± 5.0 cm/sec	0.9 ± 3.7 cm/sec	0.009*
Stroke‐affected %ΔMCAv	13.8 ± 10%	1.7 ± 5.7%	0.009*
Non‐affected resting MCAv	56.1 ± 15.0 cm/sec	75.3 ± 34.4 cm/sec	0.152
Non‐affected CVR to exercise	7.7 ± 5.7 cm/sec	0.1 ± 3.8 cm/sec	0.007*
Non‐affected %ΔMCAv	13.9 ± 11.0%	1.2 ± 4.4%	0.002*
Resting P_ET_CO_2_	35.2 ± 5.0 mmHg	33.4 ± 2.5 mmHg	0.451
Exercising P_ET_CO_2_	39.4 ± 5.3 mmHg	37.3 ± 1.5 mmHg	0.203
Resting MAP	78.5 ± 21.2 mmHg	83.7 ± 8.8 mmHg	0.289
Exercising MAP	97.9 ± 21.3 mmHg	98.1 ± 16.6 mmHg	0.984

Note

Abbreviations: %ΔMCAv, percent change in MCAv from rest to exercise; cm/sec, centimeters per second; CVR, cerebrovascular response to exercise; MAP, average mean arterial pressure; MCAv, middle cerebral artery velocity; P_ET_CO_2_, average End‐Tidal Carbon dioxide.

Values are mean ± standard deviation unless otherwise indicated. One individual selected multiple race categories.

* significant (*p* < .05).

**Figure 1:**
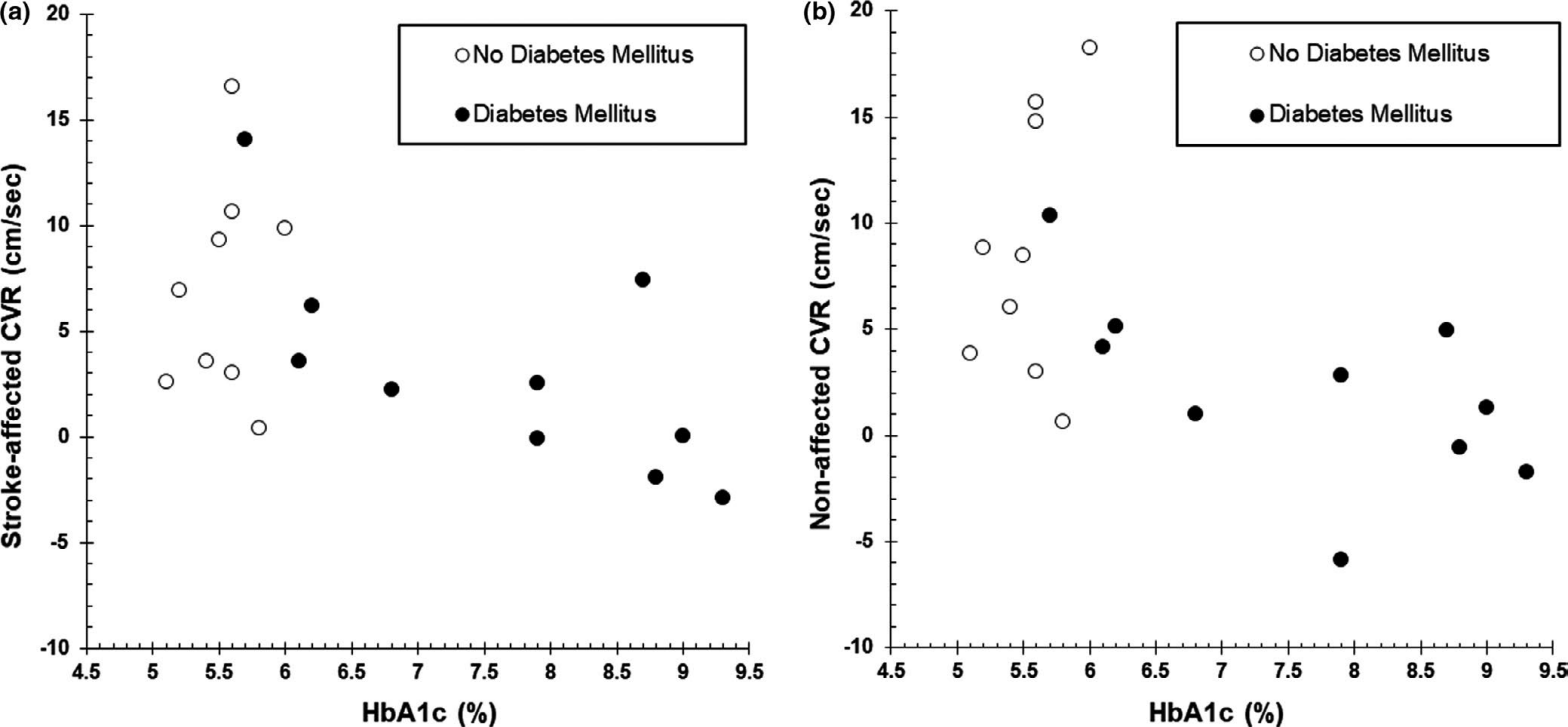
Relationship between HbA1c at the time of acute stroke and CVR to moderate‐intensity exercise at 3 months post‐stroke on the stroke‐affected (a) and non‐affected (b) side

Figure 1 shows the relationship between HbA1c and the CVR at 3 months post‐stroke for participants with (*n* = 10) and without (*n* = 9) Type 2 DM, defined as a diagnosis of Type 2 DM present in the electronic medical record at the time of stroke. For participants with Type 2 DM, preliminary analysis showed the relationship between HbA1c and CVR to be linear for both the stroke‐affected and non‐affected hemispheres. Pearson's product–moment correlation showed a strong negative correlation between HbA1c and CVR on the stroke‐affected (*r* = −0.690, *p* = .027) and approached significance for the non‐affected (*r* = −0.600, *p* = .067) side.

### 3.2 Visit 2 (6 months post‐stroke)

Data for the 12 participants who completed Visit 2 at 6 months post‐stroke were in line with findings from Visit 1. Specifically, there remained no significant between‐group differences for those with low HbA1c (<7%, *n* = 8) and high HbA1c (>7%, *n* = 4) in resting MCAv on the stroke‐affected (*p* = .683) or non‐affected (*p* = 1.000) side. Additionally, CVR to moderate‐intensity exercise remained significantly lower for the high HbA1c group than the low HbA1c group on both the stroke‐affected (*p* = .008) and non‐affected (*p* = .016) sides. Likewise, %ΔMCAv was significantly lower for the high HbA1c group than the low HbA1c group on both the stroke‐affected (*p* = .016) and non‐affected (*p* = .004) sides at 6 months post‐stroke. These data mirror Visit 1 results.

The data that support the findings of this study are available from the corresponding author upon reasonable request.

## 4 DISCUSSION

The major finding of this study was participants with high HbA1c (>7%) at the time of acute stroke demonstrated an impaired bilateral CVR to moderate‐intensity exercise 3 months later. For those who returned at 6 months post‐stroke, these same group differences remained. This suggests chronic hyperglycemia in the months prior to acute stroke, indicated by HbA1c > 7%, predicts impaired cerebral hemodynamics in the subacute and chronic phases of stroke recovery.

The ADA and ACP guidelines advise clinicians to target HbA1c < 7% in patients with DM because this has been shown to reduce vascular complications of the disease. (Qaseem et al., 2018; Targets, 2019) For individuals who do not achieve this level of glucose control, chronic hyperglycemia has been shown to cause systemic impairments in vascular function, (Qaseem et al., 2018; Targets, 2019) which likely explains the cerebrovascular dysfunction observed during exercise in the high HbA1c group in the current study. This vascular dysfunction can occur through a variety of mechanisms, the majority of which involve insults to endothelial cells. (Hadi & Suwaidi, 2007) For example, chronic hyperglycemia increases reactive oxygen species which damage endothelial cells over time. (Christen et al., 2010; Popov, 2010) Additionally, elevated blood glucose has been shown to alter endothelial cell phenotype by disrupting transcription which results in a variety of biochemical outcomes, including reduced bioavailability of nitric oxide, a molecule that normally promotes vasodilation and increases blood flow. (Addabbo et al., 2009; Hirose et al., 2010; Laughlin et al., 2008; Popov, 2010) Individuals with chronic hyperglycemia have impaired endothelium‐dependent vasodilation, as evidenced by a negative association between HbA1c and blood flow response to acetylcholine administration in the forearm. (Makimattila et al., 1996) This impaired vascular function caused by chronic hyperglycemia that has been observed peripherally extends to the cerebral vasculature. (Cui et al., 2017; Kim et al., 2011; Last et al., 2007) For example, higher HbA1c is associated with an attenuated CBF response to hypercapnia, (Last et al., 2007) and people with DM have impaired cerebrovascular autoregulation. (Kim et al., 2011).

Contrary to our hypothesis, there was no significant difference in MCAv between individuals with high or low HbA1c at rest. This contrasts with some previous reports that have shown lower resting CBF in patients with DM (Last et al., 2007) and particularly in those with poor glycemic control. (Cui et al., 2017) This could be due to differences in methodology, as these studies utilized arterial spin‐labeling magnetic resonance imaging to measure CBF, while the current study utilized TCD to assess MCAv as a surrogate of CBF. It also could reflect an adaptive mechanism unique to stroke recovery occurring in our participants at rest or simply be due to small sample size. Importantly, a physiological challenge such as exercise can reveal group differences in cerebrovascular function that may not be observed in the static, resting condition. (Sisante et al., 2019) Thus, although there were no significant group differences observed at rest, perturbations in cerebrovascular function became apparent in individuals with chronic hyperglycemia with the moderate‐intensity exercise stimulus employed in our study. Specifically, individuals with chronic hyperglycemia demonstrated an impaired CVR to exercise in both the stroke‐affected and non‐affected hemispheres at 3 and 6 months post‐stroke. Previous studies have shown the degree of chronic hyperglycemia at the time of acute stroke predicts lower functional outcomes 3 months into recovery. (Lattanzi et al., 2016; Megherbi et al., 2003; Wang et al., 2019) Additionally, chronic hyperglycemia in stroke patients has been shown to cause cerebral microvascular remodeling that impairs blood flow. (Hou et al., 2013) Our data suggest chronic hyperglycemia at the time of acute ischemic stroke may negatively affect cerebrovascular health months later, which could have implications for recovery and rehabilitation. (Billinger et al., 2014) Notably, this effect of chronic hyperglycemia on cerebrovascular function may be apparent with or without ischemic stroke. We previously reported in a subset of older adults, those with diabetes had a significantly lower CVR during moderate‐intensity exercise than those without diabetes. (Perdomo et al.,) In the present study, participants with high HbA1c demonstrated an impaired CVR to exercise in both the non‐affected and stroke‐affected hemispheres, suggesting the impact of chronic hyperglycemia may not be specific to stroke pathology. Future studies should assess whether CVR is preferentially reduced in people with a diagnosis of DM and stroke when compared to individuals with DM and no stroke pathology.

To our knowledge, we are the first to characterize the connection between chronic hyperglycemia at the time of acute stroke and cerebrovascular function during exercise months later. However, one previous study in physically active males showed individuals with DM had a lower MCAv response during a bout of high‐intensity exercise compared to participants without DM. (Kim et al., 2015) The authors proposed that the reduced MCAv response may be due to impaired cerebral vasodilatory capacity associated with the presence of DM. (Kim et al., 2015) These may be potential mechanisms contributing to the observed lower CVR in the group with chronic hyperglycemia in the present study and require further exploration. Importantly, we found that for individuals with Type 2 DM (defined as a diagnosis of Type 2 DM in the electronic medical record made by a clinician prior to stroke), HbA1c was significantly negatively correlated with CVR on the stroke‐affected side (Figure 1). These data suggest that degree of hyperglycemia is meaningful beyond a simple cutoff value of HbA1c greater or less than 7%. That is, individuals with very high HbA1c seem to have even greater cerebrovascular dysfunction than those with values only marginally above target (7%). This suggests individuals with DM can attenuate cerebrovascular dysfunction through tight, long‐term glucose control in a dose‐dependent manner, which could be important for stroke recovery.

Importantly, the exercise intensity utilized in this study requires similar effort to common activities such as brisk walking. (Ferguson,) Therefore, understanding the CVR at this intensity level could have day‐to‐day implications for brain health and inform future studies and physical activity recommendations for people post‐stroke. (Billinger et al., 2014) For example, it has been proposed that aerobic exercise mechanistically promotes beneficial adaptations of vasculature through shear stress of the vessel wall secondary to the acute increase in blood flow with exercise onset. (Lucas et al., 2015) This could suggest a blunted CVR predicts less robust improvements in cerebrovascular function with exercise if CVR reflects degree of shear stress. However, it is important to note that it remains unclear whether CVR has direct downstream clinical implications or whether it is simply an indication of poor cerebrovascular health. Overall, our results reinforce the importance of maintaining controlled chronic blood glucose level in adults, (Edelman, 1998; Valeri et al., 2004) as this may be beneficial for cerebrovascular health post‐stroke. Additionally, our data suggest that HbA1c level, which is a simple metric included in the acute stroke hospital workup, could be used by clinicians to predict cerebrovascular function during exercise in patients for at least 6 months following stroke. Thus, this routine metric could guide clinical decisions months into the recovery period.

This study has several important considerations. First, we measured MCAv as a surrogate of CBF using TCD because it is currently the only practical method for indexing CBF during exercise. (Brassard et al., 2017) This method assumes constant MCA diameter. It is unclear whether the MCA undergoes meaningful changes in diameter, (Brothers & Zhang, 2016; Hoiland et al., 2016) but alterations are likely negligible during the exercise stimulus used in this study. (Giller et al., 1993;32:^737^–^741^;; Schreiber et al., 2000; Serrador et al., 2000; Valdueza et al., 1997) Although non‐significant, differences in resting MCAv between HbA1c groups could impact CVR. However, we attempted to control for this by assessing differences in %ΔMCAv, which accounts for resting MCAv, in addition to CVR. We found the group with chronic hyperglycemia had significantly smaller %ΔMCAv, suggesting an impaired CBF response to exercise in this group independent of baseline measures. This study was limited by small sample size which did not allow for multivariate analysis to include additional potential predictors of reduced CVR, such as age and other vascular risk factors (including smoking, BMI, and hypertension). However, no significant differences in these factors (Table 1) between the high and low HbA1c groups were found. HbA1c percentage reflects average blood glucose levels over the prior 3 months, (Targets, 2019) but it does not account for cyclical changes in glucose that may occur day‐to‐day. Finally, we did not measure hematocrit or cardiac output in the participants at the time of the experimental visits, which could have impacted hemodynamics.

In the present study, we showed individuals with chronic hyperglycemia in the months immediately prior to acute ischemic stroke (as evidenced by HbA1c > 7%) have a smaller bilateral CVR and %ΔMCAv from rest to exercise measured 3 and 6 months into the post‐stroke recovery period. Additionally, we found HbA1c level at the time of acute stroke was particularly important for individuals who had a previous diagnosis of Type 2 DM, with a significant negative correlation between CVR and HbA1c for those participants. We propose this cerebrovascular dysfunction may be due to the damaging effects of chronic hyperglycemia on endothelial function, which could cause an impaired vasodilatory response during exercise. These findings highlight the importance of maintaining tight blood glucose control throughout the lifetime in order to reduce morbidity and improve cerebrovascular health.

## CONFLICT OF INTEREST

CSK, SXB, and SME report no conflicts. SAB reports a patent pending (18KU028M‐02).

## AUTHOR CONTRIBUTIONS

SME and SXB contributed to the acquisition of data and drafting/revising the manuscript. CSK contributed to the acquisition of data, analysis and interpretation of data, and drafting/revision of the manuscript. SAB contributed to the concept and design of the study, acquisition of data and funding for the project, analysis and interpretation of data, and drafting/revision of the manuscript.

### Peer Review

The peer review history for this article is available at https://publons.com/publon/10.1002/brb3.1990.

## References

[brb31990-bib-0001] Addabbo, F. , Montagnani, M. , & Goligorsky, M. S. (2009). Mitochondria and reactive oxygen species. Hypertension, 53, 885–892. 19398655 10.1161/HYPERTENSIONAHA.109.130054 PMC2716801

[brb31990-bib-0002] Alexandrov, A. V. , Sloan, M. A. , Tegeler, C. H. , Newell, D. N. , Lumsden, A. , Garami, Z. , Levy, C. R. , Wong, L. K. S. , Douville, C. , Kaps, M. , & Tsivgoulis, G. (2012). Practice standards for transcranial Doppler (TCD) ultrasound. Part II. Clinical indications and expected outcomes. Journal of Neuroimaging, 22, 215–224. 20977531 10.1111/j.1552-6569.2010.00523.x

[brb31990-bib-0003] Alexandrov, A. V. , Sloan, M. A. , Wong, L. K. , Douville, C. , Razumovsky, A. Y. , Koroshetz, W. J. , Kaps, M. , & Tegeler, C. H. (2007). Practice standards for transcranial Doppler ultrasound: Part I–test performance. Journal of Neuroimaging, 17, 11–18. 17238867 10.1111/j.1552-6569.2006.00088.x

[brb31990-bib-0004] Alwatban, M. R. , Liu, Y. , Perdomo, S. J. , Ward, J. L. , Vidoni, E. D. , Burns, J. M. , & Billinger, S. A. (2020). TCD Cerebral hemodynamic changes during moderate‐intensity exercise in older adults. Journal of Neuroimaging, 30, 76–81. 31750593 10.1111/jon.12675 PMC6954976

[brb31990-bib-0005] Billinger, S. A. , Arena, R. , Bernhardt, J. , Eng, J. J. , Franklin, B. A. , Johnson, C. M. , MacKay‐Lyons, M. , Macko, R. F. , Mead, G. E. , Roth, E. J. , Shaughnessy, M. , & Tang, A. (2014). Physical activity and exercise recommendations for stroke survivors: A statement for healthcare professionals from the American Heart Association/American Stroke Association. Stroke, 45, 2532–2553. 24846875 10.1161/STR.0000000000000022

[brb31990-bib-0006] Billinger, S. A. , Craig, J. C. , Kwapiszeski, S. J. , Sisante, J.‐F.‐ V. , Vidoni, E. D. , Maletsky, R. , & Poole, D. C. (2017). Dynamics of middle cerebral artery blood flow velocity during moderate‐intensity exercise. Journal of Applied Physiology, 122, 1125–1133. 28280106 10.1152/japplphysiol.00995.2016 PMC5451537

[brb31990-bib-0007] Billinger, S. A. , Whitaker, A. A. , Morton, A. , Kaufman, C. S. , Perdomo, S. J. , Ward, J. L. , Eickmeyer, S. M. , Bai, S. X. , Ledbetter, L. , & Abraham, M. G. (2020). Post‐stroke physical activity improves middle cerebral artery dynamic response. medRxiv, 2020, 2005, 2013.

[brb31990-bib-0008] Brassard, P. , Ferland‐Dutil, H. , Smirl, J. D. , Paquette, M. , Blanc, O. L. , Malenfant, S. , & Ainslie, P. N. (2017). Evidence for hysteresis in the cerebral pressure‐flow relationship in healthy men. American Journal of Physiology‐Heart and Circulatory Physiology, 312, H701–H704. 28130339 10.1152/ajpheart.00790.2016

[brb31990-bib-0009] Brawner, C. A. , Ehrman, J. K. , Schairer, J. R. , Cao, J. J. , & Keteyian, S. J. (2004). Predicting maximum heart rate among patients with coronary heart disease receiving beta‐adrenergic blockade therapy. American Heart Journal, 148, 910–914. 15523326 10.1016/j.ahj.2004.04.035

[brb31990-bib-0010] Brothers, R. M. , & Zhang, R. (2016). CrossTalk opposing view: The middle cerebral artery diameter does not change during alterations in arterial blood gases and blood pressure. The Journal of Physiology, 594, 4077–4079. 27010011 10.1113/JP271884 PMC4806218

[brb31990-bib-0011] Christen, A. I. , Armentano, R. L. , Miranda, A. , Graf, S. , Santana, D. B. , Zócalo, Y. , Baglivo, H. P. , & Sánchez, R. A. (2010). Arterial wall structure and dynamics in type 2 diabetes mellitus methodological aspects and pathophysiological findings. Current Diabetes Reviews, 6, 367–377. 20879975 10.2174/157339910793499146

[brb31990-bib-0012] Cui, Y. , Liang, X. , Gu, H. , Hu, Y. , Zhao, Z. , Yang, X.‐Y. , Qian, C. , Yang, Y. , & Teng, G.‐J. (2017). Cerebral perfusion alterations in type 2 diabetes and its relation to insulin resistance and cognitive dysfunction. Brain Imaging and Behavior, 11, 1248–1257. 27714551 10.1007/s11682-016-9583-9 PMC5653700

[brb31990-bib-0013] Duckworth, W. , Abraira, C. , Moritz, T. , Reda, D. , Emanuele, N. , Reaven, P. D. , Zieve, F. J. , Marks, J. , Davis, S. N. , Hayward, R. , Warren, S. R. , Goldman, S. , McCarren, M. , Vitek, M. E. , Henderson, W. G. , & Huang, G. D. (2009). Glucose control and vascular complications in veterans with type 2 diabetes. New England Journal of Medicine, 360, 129–139. 10.1056/NEJMoa0808431 19092145

[brb31990-bib-0014] Edelman, S. V. (1998). Importance of glucose control. The Medical Clinics of North America, 82, 665–687. 9706116 10.1016/s0025-7125(05)70019-5

[brb31990-bib-0015] Eknoyan, G. (2008). Adolphe Quetelet (1796–1874)–the average man and indices of obesity. Nephrology, Dialysis, Transplantation, 23, 47–51. 10.1093/ndt/gfm517 17890752

[brb31990-bib-0016] Ergul, A. , Kelly‐Cobbs, A. , Abdalla, M. , & Fagan, S. C. (2012). Cerebrovascular complications of diabetes: Focus on stroke. Endocrine, Metabolic & Immune Disorders Drug Targets, 12, 148–158. 10.2174/187153012800493477 PMC3741336 22236022

[brb31990-bib-0017] Ferguson, B. (2014). ACSM’s guidelines for exercise testing and prescription. The Journal of the Canadian Chiropractic Association, 58, 328.

[brb31990-bib-0018] Fisher, J. P. , Ogoh, S. , Young, C. N. , Raven, P. B. , & Fadel, P. J. (1985). Regulation of middle cerebral artery blood velocity during dynamic exercise in humans: Influence of aging. Journal of Applied Physiology (Bethesda, Md), 2008(105), 266–273. 10.1152/japplphysiol.00118.2008 PMC4073909 18467548

[brb31990-bib-0019] Giller, C. A. , Bowman, G. , Dyer, H. , Mootz, L. , & Krippner, W. (1993). Cerebral arterial diameters during changes in blood pressure and carbon dioxide during craniotomy. Neurosurgery, 32, 737–741. 8492848

[brb31990-bib-0020] Hadi, H. A. R. , & Suwaidi, J. A. (2007). Endothelial dysfunction in diabetes mellitus. Vascular Health and Risk Management, 3, 853–876. 18200806 PMC2350146

[brb31990-bib-0021] Hemmingsen, B. , Lund, S. S. , Gluud, C. , Vaag, A. , Almdal, T. , Hemmingsen, C. , & Wetterslev, J. (2011). Intensive glycaemic control for patients with type 2 diabetes: Systematic review with meta‐analysis and trial sequential analysis of randomised clinical trials. BMJ (Clinical Research ed), 343, d6898. 10.1136/bmj.d6898 PMC3223424 22115901

[brb31990-bib-0022] Hirose, A. , Tanikawa, T. , Mori, H. , Okada, Y. , & Tanaka, Y. (2010). Advanced glycation end products increase endothelial permeability through the RAGE/Rho signaling pathway. FEBS Letters, 584, 61–66. 19944695 10.1016/j.febslet.2009.11.082

[brb31990-bib-0023] Hoiland, R. L. , & Ainslie, P. N. (2016). Rebuttal from Ryan L. Hoiland and Philip N. Ainslie. The Journal of Physiology, 594, 4081. 27010009 10.1113/JP272241 PMC4967744

[brb31990-bib-0024] Hou, Q. , Zuo, Z. , Michel, P. , Zhang, Y. , Eskandari, A. , Man, F. , Gao, Q. , Johnston, K. C. , & Wintermark, M. (2013). Influence of chronic hyperglycemia on cerebral microvascular remodeling: An in vivo study using perfusion computed tomography in acute ischemic stroke patients. Stroke, 44, 3557–3560. 24029632 10.1161/STROKEAHA.113.003150

[brb31990-bib-0025] Huber, J. D. (2008). Diabetes, cognitive function, and the blood‐brain barrier. Current Pharmaceutical Design, 14, 1594–1600. 18673200 10.2174/138161208784705441

[brb31990-bib-0026] Jansen, J. F. A. , van Bussel, F. C. G. , van de Haar, H. J. , van Osch, M. J. P. , Hofman, P. A. M. , van Boxtel, M. P. J. , van Oostenbrugge, R. J. , Schram, M. T. , Stehouwer, C. D. A. , Wildberger, J. E. , & Backes, W. H. (2016). Cerebral blood flow, blood supply, and cognition in Type 2 Diabetes Mellitus. Scientific Reports, 6, 10. 27920431 10.1038/s41598-016-0003-6 PMC8276879

[brb31990-bib-0027] Kadoi, Y. , Saito, S. , Goto, F. (2004). The effect of diabetes on the interrelationship between jugular venous oxygen saturation responsiveness to phenylephrine infusion and cerebrovascular carbon dioxide reactivity. Anesthesia and Analgesia, 99, 325–331. 15271699 10.1213/01.ANE.0000132693.69567.70

[brb31990-bib-0028] Kaufman, C. S. , Bai, S. X. , Ward, J. L. , Eickmeyer, S. M. , & Billinger, S. A. (2019). Middle cerebral artery velocity dynamic response profile during exercise is attenuated following multiple ischemic strokes: A case report. Physiological Reports, 7, e14268. 31691542 10.14814/phy2.14268 PMC6831948

[brb31990-bib-0029] Kempf, K. S. W. , Alicen, A. W. , Lui, Y. , Witte, E. , Perdomo, S. J. , Ward, J. L. , Eickmeyer, S. , Ledbetter, L. , Abraham, M. , & Billinger, S. A. (2019). The effect of stroke on middle cerebral artery blood flow velocity dynamics during exercise. Journal of Neurologic Physical Therapy, 43(4), 212–219. 31449179 10.1097/NPT.0000000000000289 PMC6744289

[brb31990-bib-0030] Kernan, W. N. , Ovbiagele, B. , Black, H. R. , Bravata, D. M. , Chimowitz, M. I. , Ezekowitz, M. D. , Fang, M. C. , Fisher, M. , Furie, K. L. , Heck, D. V. , Johnston, S. C. C. , Kasner, S. E. , Kittner, S. J. , Mitchell, P. H. , Rich, M. W. , Richardson, D. J. , Schwamm, L. H. , & Wilson, J. A. (2014). Guidelines for the prevention of stroke in patients with stroke and transient ischemic attack: A guideline for healthcare professionals from the American Heart Association/American Stroke Association. Stroke, 45, 2160–2236. 24788967 10.1161/STR.0000000000000024

[brb31990-bib-0031] Kim, Y. S. , Davis, S. C. , Truijen, J. , Stok, W. J. , Secher, N. H. , & van Lieshout, J. J. (2011). Intensive blood pressure control affects cerebral blood flow in type 2 diabetes mellitus patients. Hypertension, 57, 738–745. 21357278 10.1161/HYPERTENSIONAHA.110.160523

[brb31990-bib-0032] Kim, Y.‐S. , Seifert, T. , Brassard, P. , Rasmussen, P. , Vaag, A. , Nielsen, H. B. , Secher, N. H. , & van Lieshout, J. J. (2015). Impaired cerebral blood flow and oxygenation during exercise in type 2 diabetic patients. Physiological Reports, 3, e12430. 26109188 10.14814/phy2.12430 PMC4510631

[brb31990-bib-0033] Last, D. , Alsop, D. C. , Abduljalil, A. M. , Marquis, R. P. , de Bazelaire, C. , Hu, K. , Cavallerano, J. , & Novak, V. (2007). Global and regional effects of type 2 diabetes on brain tissue volumes and cerebral vasoreactivity. Diabetes Care, 30, 1193–1199. 17290035 10.2337/dc06-2052 PMC2031924

[brb31990-bib-0034] Lattanzi, S. , Bartolini, M. , Provinciali, L. , & Silvestrini, M. (2016). Glycosylated hemoglobin and functional outcome after acute ischemic stroke. Journal of Stroke and Cerebrovascular Diseases, 25, 1786–1791. 27103269 10.1016/j.jstrokecerebrovasdis.2016.03.018

[brb31990-bib-0035] Laughlin, M. H. , Newcomer, S. C. , & Bender, S. B. (2008). Importance of hemodynamic forces as signals for exercise‐induced changes in endothelial cell phenotype. Journal of Applied Physiology, 104, 588–600. 18063803 10.1152/japplphysiol.01096.2007 PMC3289055

[brb31990-bib-0036] Lucas, S. J. E. , Cotter, J. D. , Brassard, P. , & Bailey, D. M. (2015). High‐intensity interval exercise and cerebrovascular health: Curiosity, cause, and consequence. Journal of Cerebral Blood Flow and Metabolism, 35, 902–911. 25833341 10.1038/jcbfm.2015.49 PMC4640257

[brb31990-bib-0037] Makimattila, S. , Virkamaki, A. , Groop, P. H. , Cockcroft, J. , Utriainen, T. , Fagerudd, J. , & Yki‐Järvinen, H. (1996). Chronic hyperglycemia impairs endothelial function and insulin sensitivity via different mechanisms in insulin‐dependent diabetes mellitus. Circulation, 94, 1276–1282. 8822980 10.1161/01.cir.94.6.1276

[brb31990-bib-0038] Megherbi, S. E. , Milan, C. , Minier, D. , Couvreur, G. , Osseby, G.‐V. , Tilling, K. , Carlo, A. D. , Inzitari, D. , Wolfe, C. D. A. , Moreau, T. , & Giroud, M. , European BIOMED Study of Stroke Care Group (2003). Association between diabetes and stroke subtype on survival and functional outcome 3 months after stroke: Data from the European BIOMED Stroke Project. Stroke, 34, 688–694. 12624292 10.1161/01.STR.0000057975.15221.40

[brb31990-bib-0039] Novak, V. , Last, D. , Alsop, D. C. , Abduljalil, A. M. , Hu, K. , Lepicovsky, L. , Cavallerano, J. , & Lipsitz, L. A. (2006). Cerebral blood flow velocity and periventricular white matter hyperintensities in type 2 diabetes. Diabetes Care, 29, 1529–1534. 16801574 10.2337/dc06-0261 PMC1978169

[brb31990-bib-0040] Patel, A. , MacMahon, S. , Chalmers, J. , Neal, B. , Billot, L. , Woodward, M. , Marre, M. , Cooper, M. , Glasziou, P. , Grobbee, D. , Hamet, P. , Harrap, S. , Heller, S. , Liu, L. , Mancia, G. , Mogensen, C. E. , Pan, C. , Poulter, N. , Rodgers, A. , … Travert, F. (2008). Intensive blood glucose control and vascular outcomes in patients with type 2 diabetes. New England Journal of Medicine, 358, 2560–2572. 10.1056/NEJMoa0802987 18539916

[brb31990-bib-0041] Perdomo, S. J. , Ward, J. , Liu, Y. , Vidoni, E. D. , Sisante, J. F. , Kirkendoll, K. , Burns, J. M. , & Billinger, S. A. (2019). Cardiovascular disease risk is associated with middle cerebral artery blood flow velocity in older adults. Cardiopulmonary Physical Therapy Journal, 31(2), 38–46. 10.1097/cpt.0000000000000110 PMC7580865 33100924

[brb31990-bib-0042] Popov, D. (2010). Endothelial cell dysfunction in hyperglycemia: Phenotypic change, intracellular signaling modification, ultrastructural alteration, and potential clinical outcomes. International Journal of Diabetes Mellitus, 2, 189–195.

[brb31990-bib-0043] Qaseem, A. , Wilt, T. J. , Kansagara, D. , Horwitch, C. , Barry, M. J. , & Forciea, M. A. , Clinical Guidelines Committee of the American College of Physicians (2018). Hemoglobin A1c targets for glycemic control with pharmacologic therapy for nonpregnant adults with type 2 diabetes mellitus: A guidance statement update from the american college of physicians. Annals of Internal Medicine, 168, 569–576. 29507945 10.7326/M17-0939

[brb31990-bib-0044] Robertson, A. D. , Atwi, S. , Kostoglou, K. , Verhoeff, N. P. L. G. , Oh, P. I. , Mitsis, G. D. , Marzolini, S. , & MacIntosh, B. J. (2019). Cerebrovascular pulsatility during rest and exercise reflects hemodynamic impairment in stroke and cerebral small vessel disease. Ultrasound in Medicine and Biology, 45, 3116–3127. 31570171 10.1016/j.ultrasmedbio.2019.08.019

[brb31990-bib-0045] Schreiber, S. J. , Gottschalk, S. , Weih, M. , Villringer, A. , & Valdueza, J. M. (2000). Assessment of blood flow velocity and diameter of the middle cerebral artery during the acetazolamide provocation test by use of transcranial Doppler sonography and MR imaging. AJNR American Journal of Neuroradiology, 21, 1207–1211. 10954270 PMC8174897

[brb31990-bib-0046] Serrador, J. M. , Picot, P. A. , Rutt, B. K. , Shoemaker, J. K. , & Bondar, R. L. (2000). MRI measures of middle cerebral artery diameter in conscious humans during simulated orthostasis. Stroke, 31, 1672–1678. 10.1161/01.STR.31.7.1672 10884472

[brb31990-bib-0047] Sisante, J. V. , Vidoni, E. D. , Kirkendoll, K. , Ward, J. , Liu, Y. , Kwapiszeski, S. , Maletsky, R. , Burns, J. M. , & Billinger, S. A. (2019). Blunted cerebrovascular response is associated with elevated beta‐amyloid. Journal of Cerebral Blood Flow and Metabolism, 39, 89–96. 28914134 10.1177/0271678X17732449 PMC6311677

[brb31990-bib-0048] Targets, G. (2019). Standards of medical care in diabetes‐2019. Diabetes Care, 42, S61–S70. 30559232 10.2337/dc19-S006

[brb31990-bib-0049] Valdueza, J. M. , Balzer, J. O. , Villringer, A. , Vogl, T. J. , Kutter, R. , & Einhäupl, K. M. (1997). Changes in blood flow velocity and diameter of the middle cerebral artery during hyperventilation: Assessment with MR and transcranial Doppler sonography. AJNR American Journal of Neuroradiology, 18, 1929–1934. 9403456 PMC8337363

[brb31990-bib-0050] Valeri, C. , Pozzilli, P. , & Leslie, D. (2004). Glucose control in diabetes. Diabetes/metabolism Research and Reviews, 20(Suppl 2), S1–S8. 15551299 10.1002/dmrr.512

[brb31990-bib-0051] Wang, H. , Cheng, Y. , Chen, S. , Li, X. , Zhu, Z. , & Zhang, W. (2019). Impact of elevated hemoglobin A1c levels on functional outcome in patients with acute ischemic stroke. Journal of Stroke and Cerebrovascular Diseases, 28, 470–476. 30415918 10.1016/j.jstrokecerebrovasdis.2018.10.026

[brb31990-bib-0052] Ward, J. L. , Craig, J. C. , Liu, Y. , Vidoni, E. D. , Maletsky, R. , Poole, D. C. , & Billinger, S. A. (2018). Effect of healthy aging and sex on middle cerebral artery blood velocity dynamics during moderate‐intensity exercise. American Journal of Physiology Heart and Circulatory Physiology, 315, H492–H501. 29775407 10.1152/ajpheart.00129.2018 PMC6172645

